# Regional convergence and spatial dynamics of physician workforce distribution across regions in Türkiye (2008–2023)

**DOI:** 10.1186/s12913-026-14519-w

**Published:** 2026-04-24

**Authors:** Ahmet Düha Koç

**Affiliations:** Department of Health Management, Faculty of Health Sciences, Karabük University, Karabük, Türkiye

**Keywords:** Physician distribution, Regional inequality, Spatial analysis, Convergence, Health workforce, Türkiye, Spatial econometrics, Health services research

## Abstract

**Background:**

Unequal distribution of physicians remains a persistent challenge for health systems, particularly in countries with marked regional disparities. While increases in the overall physician workforce may improve aggregate service capacity, it is unclear whether such growth translates into balanced regional access to care. This study examines long-term regional dynamics in physician distribution in Türkiye to assess whether historically underserved regions have narrowed gaps in physician-related service capacity.

**Methods:**

A longitudinal ecological study was conducted using panel data from 26 regions in Türkiye over the period 2008–2023. Physician density (per 100,000 population) was used as a proxy for regional health service capacity. Convergence was assessed using two-way fixed effects models with Driscoll–Kraay standard errors. To address potential dynamic panel bias, a dynamic panel model was estimated as a robustness check. Spatial dependence and spillover effects were analysed using spatial econometric models. Dispersion trends were also examined over time.

**Results:**

Physician density increased substantially across all regions over the study period. The results indicate statistically significant convergence, suggesting that regions with initially lower physician density experienced relatively faster growth. However, convergence was incomplete, and substantial regional differences persisted. Economic capacity and health infrastructure were positively associated with physician availability, while spatial spillover effects were limited. Dispersion analysis confirmed a reduction in regional inequality over time, although regional heterogeneity remained.

**Conclusion:**

Physician supply in Türkiye has expanded and regional disparities have narrowed, but convergence has been partial and uneven. National workforce growth alone appears insufficient to eliminate persistent regional imbalances in service capacity. Policies that combine workforce expansion with targeted regional development and infrastructure investment may be necessary to achieve more equitable health service provision.

**Supplementary Information:**

The online version contains supplementary material available at 10.1186/s12913-026-14519-w.

## Introduction

The equitable provision of health services is a core objective of modern health systems and a prerequisite for achieving universal health coverage. Central to this objective is the availability and geographic distribution of the physician workforce, which directly determines health service capacity, accessibility, and continuity of care. Despite sustained global increases in physician numbers, substantial regional disparities in physician availability persist within countries, limiting effective service delivery and undermining health system performance [1–3].

Evidence from both high-income and middle-income countries consistently shows that physician maldistribution is more resistant to policy intervention than overall workforce shortages. Regions with lower physician density often experience reduced access to essential services, longer waiting times, higher unmet need, and poorer health outcomes, even when national supply levels appear adequate [4–6]. These disparities are particularly pronounced in countries with large geographic, socioeconomic, and infrastructural heterogeneity, where uniform workforce policies may fail to address region-specific constraints [7, 8].

From a health services perspective, physician distribution reflects not only labour market dynamics but also the spatial organisation of service delivery systems. Physicians tend to cluster in regions offering better economic opportunities, professional networks, infrastructure, and quality of life, reinforcing existing regional advantages and creating persistent service capacity gaps elsewhere [9–11]. As a result, regional health service performance becomes uneven, with disadvantaged areas facing cumulative challenges related to access, continuity, and quality of care [12].

In response to these challenges, international organisations such as the World Health Organization and the Organisation for Economic Co-operation and Development have emphasised the need for regionally sensitive health workforce planning that integrates economic, infrastructural, and demographic considerations into allocation strategies [13, 14]. However, much of the existing empirical evidence remains descriptive, focusing on cross-sectional disparities rather than examining how regional physician supply evolves over time and whether lagging regions are catching up with more advantaged ones [15].

Convergence theory, originally developed in regional economics, provides a dynamic framework for assessing whether disparities across regions are narrowing over time. Applied to health services, convergence analysis allows researchers to evaluate whether regions with initially lower physician density experience faster growth, thereby reducing gaps in service capacity [16]. β-convergence, in particular, captures the speed at which less-served regions catch up with better-served ones, offering an interpretable metric for long-term system performance and workforce planning effectiveness [17].

Several studies have applied convergence concepts to health-related resources, including hospital beds, health expenditure, and physician supply, with mixed findings across countries. While some European regions exhibit gradual convergence in physician density, others display persistent divergence, suggesting that national policies alone may be insufficient to overcome structural regional disadvantages [18]. Importantly, convergence in national averages does not necessarily translate into equitable service delivery if improvements remain geographically concentrated [19].

Spatial dependence further complicates regional workforce dynamics. Health service capacity in one region may be influenced by neighbouring regions through professional mobility, referral networks, and shared infrastructure. Ignoring such spatial interactions risks biased estimates and incomplete policy interpretation. Spatial econometric approaches enable the identification of geographic clustering and spillover effects that shape regional service capacity beyond administrative boundaries [20].

Türkiye provides a particularly relevant case for examining regional dynamics in physician distribution and health service capacity. Since the early 2000s, the country has implemented comprehensive health system reforms, including expansion of insurance coverage, large-scale hospital investments, and substantial increases in medical education capacity. These reforms have contributed to a marked rise in overall physician density and improvements in service utilisation at the national level [21].

Despite these gains, regional disparities in physician availability remain pronounced. Western and metropolitan regions consistently exhibit higher physician density and service capacity, while eastern and southeastern regions continue to face relative shortages, particularly in specialised care [22, 23]. These patterns persist despite centrally administered workforce policies, suggesting that structural regional factors continue to shape health service capacity [24].

Existing studies on physician distribution in Türkiye have largely relied on cross-sectional analyses or limited time horizons, restricting insight into long-term regional dynamics and policy effectiveness [22–24]. Moreover, few studies have simultaneously examined convergence processes and spatial interactions using longitudinal regional data, leaving an important gap in the health services literature.

Against this background, the present study examines long-term regional dynamics in physician workforce distribution across Türkiye’s 26 NUTS-2 regions from 2008 to 2023, framing physician density as an indicator of regional health service capacity. By combining β-convergence analysis with spatial econometric methods, the study assesses whether regional disparities in physician supply are narrowing over time and whether spatial spillovers contribute to the diffusion of physician availability across regions.

By adopting a longitudinal and spatially explicit approach, this study contributes to the health services research literature by moving beyond static descriptions of inequality and offering policy-relevant insights for designing regionally responsive health workforce strategies in middle-income countries.

## Methods

### Study design

This study employed a longitudinal ecological design based on regional-level panel data to examine temporal and spatial dynamics in physician workforce distribution as an indicator of health service capacity. The analytical framework combined panel-data econometric techniques with spatial analysis to assess both convergence processes and geographic interdependence in physician availability across regions, an approach commonly adopted in regional health services research [25–27].

### Study setting and units of analysis

The study was conducted at the NUTS-2 regional level in Türkiye, comprising 26 regions defined under the European Union’s Nomenclature of Territorial Units for Statistics, which provides a harmonised territorial classification for regional analysis and international comparability [28]. These regions represent the primary administrative scale for regional development planning and health policy analysis. The observation period covered 2008–2023, yielding a balanced panel of 416 region–year observations (26 regions × 16 years).

### Data sources

Data were obtained exclusively from publicly available and official secondary sources to ensure transparency and reproducibility. Annual regional physician counts and hospital bed data were retrieved from the health statistics yearbooks [29]. Population data used to calculate physician density were obtained from the Turkish Statistical Institute [30]. Regional gross domestic product (GDP) per capita, adjusted for purchasing power parity (PPP), was sourced from the OECD Regional Database [31]. All datasets were harmonised using consistent regional identifiers and calendar years in line with best practices for panel data construction.

### Variables

#### Outcome variable

The primary outcome variable was physician density, defined as the number of practising physicians per 100,000 population in each region and year. Physician density is widely used in the literature as a proxy for regional health service capacity and access to medical care [32, 33].

#### Explanatory variables

Four regional-level covariates were included based on theoretical relevance and prior empirical evidence:

1. **Economic capacity**: GDP per capita (PPP-adjusted, constant prices), capturing regional economic conditions associated with labour market attractiveness and demand for health services [34].
2. **Health infrastructure**: Hospital beds per 100 population, reflecting physical service capacity and institutional readiness to support physician practice [35].
3. **Educational context**: Female tertiary education attainment (%), used as a proxy for regional human capital and broader social infrastructure influencing workforce retention and service utilisation patterns [36].
4. **Demographic structure**: Regional age composition (share of population aged ≥ 65), included to account for differences in healthcare demand associated with ageing populations, which may influence physician distribution patterns and service needs.

All continuous variables were log-transformed to reduce skewness and facilitate elasticity-based interpretation, except proportion variables, which were included in level form to preserve interpretability.

### Econometric analysis

#### β-convergence analysis

To assess whether regions with initially lower physician density experienced faster subsequent growth, β-convergence models were estimated using two-way fixed effects (TWFE) regression, a standard approach in regional convergence analysis [37, 38]. This framework controls for unobserved, time-invariant regional characteristics and common national shocks affecting all regions simultaneously. In addition, lagged dependent variables were included to capture dynamic adjustment processes in physician distribution over time.

#### Baseline model

$$\begin{aligned}\rm \Delta\:ln(Physicians\_it)=&\alpha + \beta\:ln(Physicians\_i,t-1)\cr&+\gamma X\_it + \mu\_i + \lambda\_t + \varepsilon\_it\:\end{aligned}$$

#### Dynamic panel robustness (system GMM)

To address potential bias associated with including a lagged dependent variable in a fixed effects framework (Nickell bias), an additional system Generalized Method of Moments (System GMM) estimator was implemented. This approach, based on the Arellano–Bover/Blundell–Bond framework, uses internal instruments derived from lagged levels and differences of the dependent variable.

The validity of the instruments was evaluated using the Hansen test of overidentifying restrictions and the Arellano–Bond test for second-order autocorrelation (AR(2)). The GMM specification was used as a robustness check to confirm whether convergence results remained consistent when potential endogeneity and dynamic panel bias were explicitly addressed.

To address heteroskedasticity, serial correlation, and cross-sectional dependence commonly observed in regional panel data, Driscoll–Kraay robust standard errors were applied [39].

#### Convergence speed and half-life

The speed of convergence was quantified by calculating the implied half-life, defined as the number of years required for half of the initial disparity in physician density to be eliminated. This metric is frequently used in convergence studies to provide an intuitive interpretation of adjustment dynamics [37].

The half-life (HL) was calculated using the standard formulation:

$$\rm HL = ln(2) / |\beta|$$

where β represents the estimated convergence coefficient. Given the relatively rapid estimated convergence, this metric is interpreted cautiously as reflecting relative catch-up dynamics rather than complete structural equalisation across regions.

Detailed half-life sensitivity results are provided in the Supplementary Material (Table S1).

Additional descriptive and robustness results are provided in the Supplementary Material (Tables S1–S10).

### Spatial analysis

#### Spatial autocorrelation

Global spatial autocorrelation in physician density was assessed using Moran’s I statistic, a widely used measure for detecting spatial clustering in regional data [40].

Local Indicators of Spatial Association (LISA) analysis was conducted to examine local spatial clustering patterns and identify potential regional clusters of high and low physician density. Detailed LISA results are presented in the Supplementary Material.

#### Spatial Durbin Model

To explicitly model spatial dependence and spillover effects, Spatial Durbin Models (SDM) were estimated. The SDM specification incorporates both spatially lagged dependent variables and spatially lagged covariates, allowing the identification of direct (within-region) and indirect (spillover) effects [41, 42].

The SDM specification can be expressed as:

$$\begin{aligned} \rm y\_it =&\rho W y\_it + X\_it\:\beta + WX\_it\:\theta\cr&+ \mu\_i + \lambda\_t+ \varepsilon\_it\end{aligned}$$

where W denotes the spatial weights matrix, ρ captures spatial dependence, and θ represents spatial spillover effects.

The SDM was preferred over more restrictive alternatives such as the Spatial Autoregressive (SAR) and Spatial Error Model (SEM) due to its ability to jointly estimate endogenous interaction effects and exogenous spillovers, consistent with recent applications in regional health services research [41].

#### σ-convergence

To complement the β-convergence analysis, σ-convergence was assessed by examining changes in dispersion in physician density across regions over time. Dispersion was measured using the coefficient of variation (CV), calculated as the ratio of the standard deviation to the mean.

A declining trend in the CV indicates a reduction in regional disparities, while a stable or increasing trend suggests persistent inequality. Both unweighted and population-weighted measures were examined to ensure robustness.

#### Robustness and supplementary analyses

Several robustness checks were conducted to assess the stability of findings. Alternative model specifications were estimated, including exclusion of metropolitan outliers and population-weighted regressions. Additional robustness checks included alternative spatial weight matrices and dynamic panel specifications. Rank-mobility analysis was used to examine changes in regional physician density rankings over time, complementing convergence estimates by capturing relative positional mobility across regions [38].

#### Software and reproducibility

All analyses were conducted using R (version 4.4.3). Panel models were estimated using the plm and fixest packages, while spatial analyses were performed using spdep and splm, consistent with contemporary practice in applied spatial econometrics and health services research [42, 43]. Data processing and visualisation utilised dplyr, ggplot2, sf, and tmap. Dynamic panel models were implemented using the pgmm function within the plm package. All analytical scripts are available from the corresponding author upon reasonable request, in line with reproducibility standards.

Additional supplementary analyses and tables are provided in the Supplementary Material (Tables S1–S10).

## Results

### Descriptive trends in physician supply

**Fig. 1:**
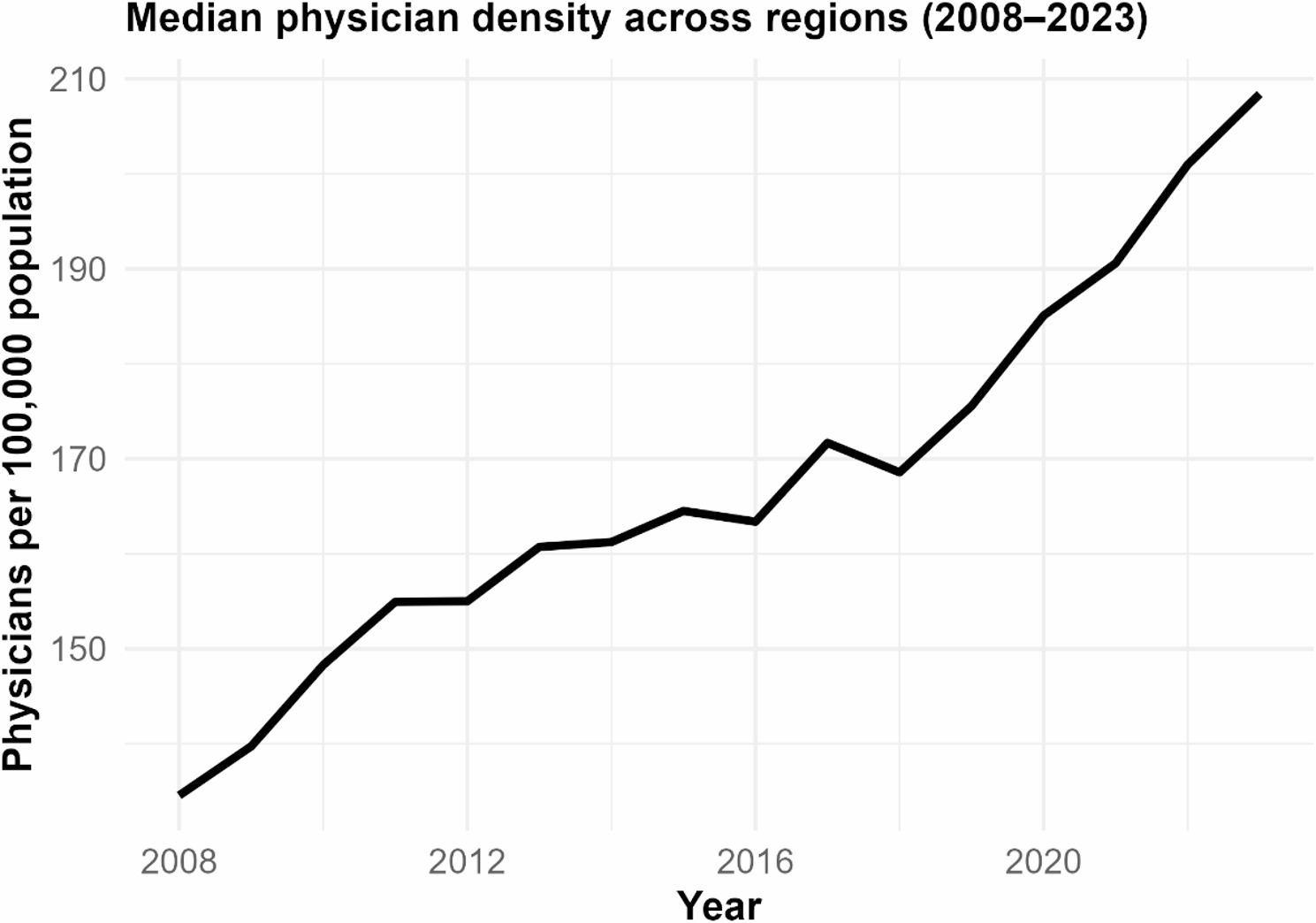
Trends in median physician density across regions, 2008–2023. Physician density is expressed as the number of physicians per 100,000 population. The figure displays the median value across 26 NUTS-2 regions for each year, illustrating the overall temporal trend in regional health service capacity

Between 2008 and 2023, physician density increased steadily across all 26 NUTS-2 regions in Türkiye, indicating an overall expansion in health service capacity nationwide. The national median physician density rose from approximately 130 physicians per 100,000 population in 2008 to over 210 in 2023. Despite this upward trend, substantial regional variation persisted throughout the study period, with western and metropolitan regions consistently exhibiting higher physician availability than eastern and southeastern regions (Fig. 1).

To complement this descriptive assessment, dispersion in physician density across regions was examined using the coefficient of variation (CV). The CV declined over time, indicating a gradual reduction in regional disparities, although the decline was not uniform across the study period. This pattern suggests partial σ-convergence, with persistent heterogeneity in regional service capacity.

**Fig. 2:**
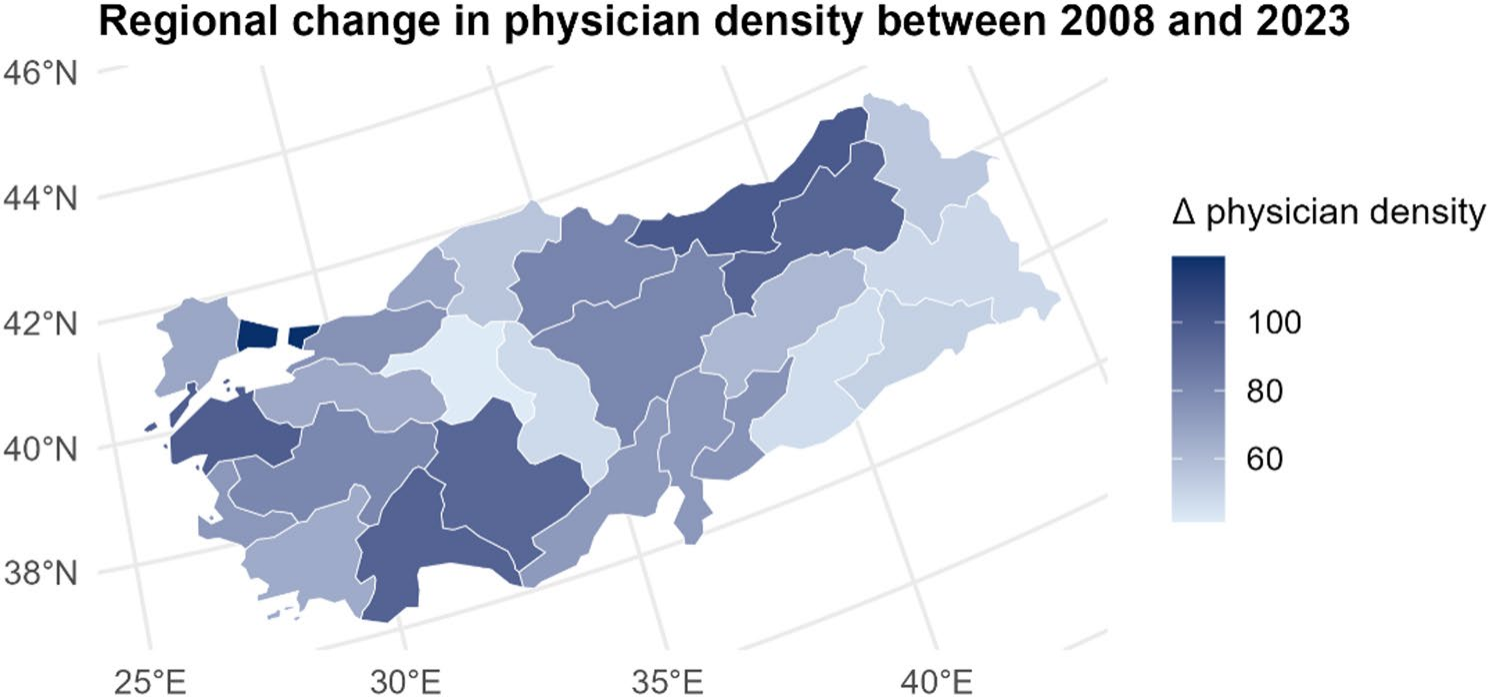
Regional change in physician density between 2008 and 2023. The map illustrates the absolute change in physician density (physicians per 100,000 population) across NUTS-2 regions. Darker shades indicate larger increases in physician density over the study period

The spatial distribution of changes in physician density revealed pronounced regional heterogeneity. Regions located in Central Anatolia and the western part of the country experienced larger absolute increases in physician supply, while several eastern regions showed comparatively modest gains (Fig. 2). These patterns suggest that national workforce growth has not translated uniformly into balanced regional service capacity.

### β-convergence in regional physician supply

Results from the baseline two-way fixed effects model with Driscoll–Kraay robust standard errors demonstrated statistically significant β-convergence in physician density across regions (Table 1). The estimated coefficient for lagged physician density was negative and highly significant, indicating that regions with initially lower physician availability experienced faster subsequent growth in physician supply.

**Table 1:** Results from panel regression models examining convergence in physician density

Variables	Model 1: TWFE	Model 2: Robust (Excl. TR10)	Model 3: System GMM
Lagged physician density	-0.424***	-0.416***	-0.410***
	(0.078)	(0.086)	(0.082)
GDP per capita	0.00000216***	0.00000226***	0.00000210***
	(0.00000064)	(0.00000057)	(0.00000060)
Education (female tertiary)	0.526	0.370	0.410
	(0.572)	(1.793)	(0.980)
Hospital beds	0.347***	0.296***	0.320***
	(0.112)	(0.085)	(0.100)

Observations: 416

Region FE: Yes

Year FE: Yes

Notes: Robust standard errors in parentheses. *** *p* < 0.01, ** *p* < 0.05, * *p* < 0.1

The implied convergence speed corresponded to a half-life of approximately 1.6 years, suggesting that regional disparities in physician-related service capacity have narrowed over the study period. However, this estimate should be interpreted cautiously, as it reflects relative catch-up dynamics rather than rapid structural equalisation across regions.

To address potential dynamic panel bias and endogeneity, a system GMM model was estimated as a robustness check. The results confirmed the presence of convergence, with the lagged dependent variable remaining negative and statistically significant. The magnitude of the coefficient was comparable to the baseline estimates, indicating that the convergence findings are not driven by estimation bias.

This finding remained robust across alternative model specifications, including models excluding metropolitan outliers and population-weighted estimations (Table 1).

Economic capacity and health infrastructure were consistently associated with higher physician density. Regional GDP per capita and hospital beds per 100 population both showed positive and statistically significant associations with physician availability, while educational attainment did not demonstrate a stable independent relationship across specifications.

The inclusion of demographic structure (age composition) further improved model specification by accounting for variation in healthcare demand across regions, although its effect varied across model specifications.

### Robustness and alternative specifications

Sensitivity analyses confirmed the stability of the convergence results. Excluding Istanbul (TR10), which represents an extreme outlier in physician density, did not materially alter the magnitude or significance of the β-convergence coefficient.

Similarly, alternative specifications yielded comparable half-life estimates, ranging between 1.4 and 1.8 years, reinforcing confidence in the robustness of the convergence dynamics (Table 1).

Additional robustness checks using dynamic panel estimation and alternative spatial weight matrices produced consistent results, further supporting the stability of the findings.

### Spatial dependence and spillover effects

**Fig. 3:**
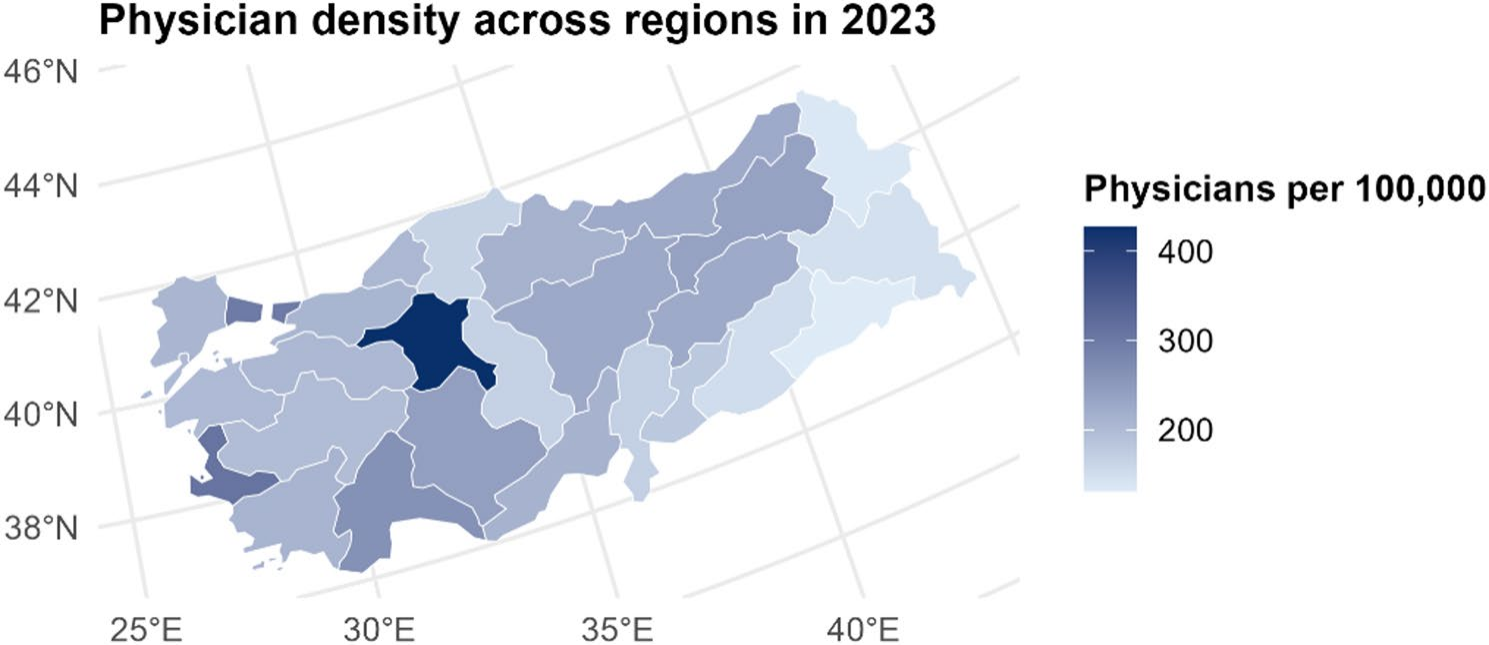
Physician density across regions in 2023. The map presents the spatial distribution of physician density (physicians per 100,000 population) across NUTS-2 regions. Darker shades represent higher physician density levels

Global Moran’s I statistics indicated weak and statistically insignificant spatial autocorrelation, suggesting limited geographic clustering of physician-related service capacity (Fig. 3).

This pattern implies that improvements in physician availability have been largely region-specific rather than strongly diffusing across neighbouring regions.

Spatial Durbin Model estimates further supported this interpretation (Table 2). Direct effects of regional economic capacity and hospital infrastructure on physician density were strong and statistically significant.

**Table 2:** Spatial Durbin Model estimates and decomposition of direct and spillover effects

Variables	Direct Effect	Indirect Effect	Total Effect
GDP per capita	0.000017***	0.000002	0.000019***
Education	-2.21	-11.07*	-13.41*
Hospital beds	2.27***	-0.67	1.59

Spatial parameter (ρ): 0.233 (*p* = 0.332)

Notes: Direct and indirect effects derived from SDM impact decomposition.

*** *p* < 0.01, ** *p* < 0.05, * *p* < 0.1

The spatial autoregressive coefficient was positive but not statistically significant, indicating limited spatial dependence in physician distribution.

In contrast, indirect (spillover) effects transmitted through neighbouring regions were small and generally not statistically significant.

Impact decomposition confirmed that within-region effects dominated over spillover effects (Table 2), suggesting that local economic and infrastructural conditions play a substantially greater role in shaping physician availability than interregional diffusion mechanisms.

### Regional rank mobility

**Fig. 4:**
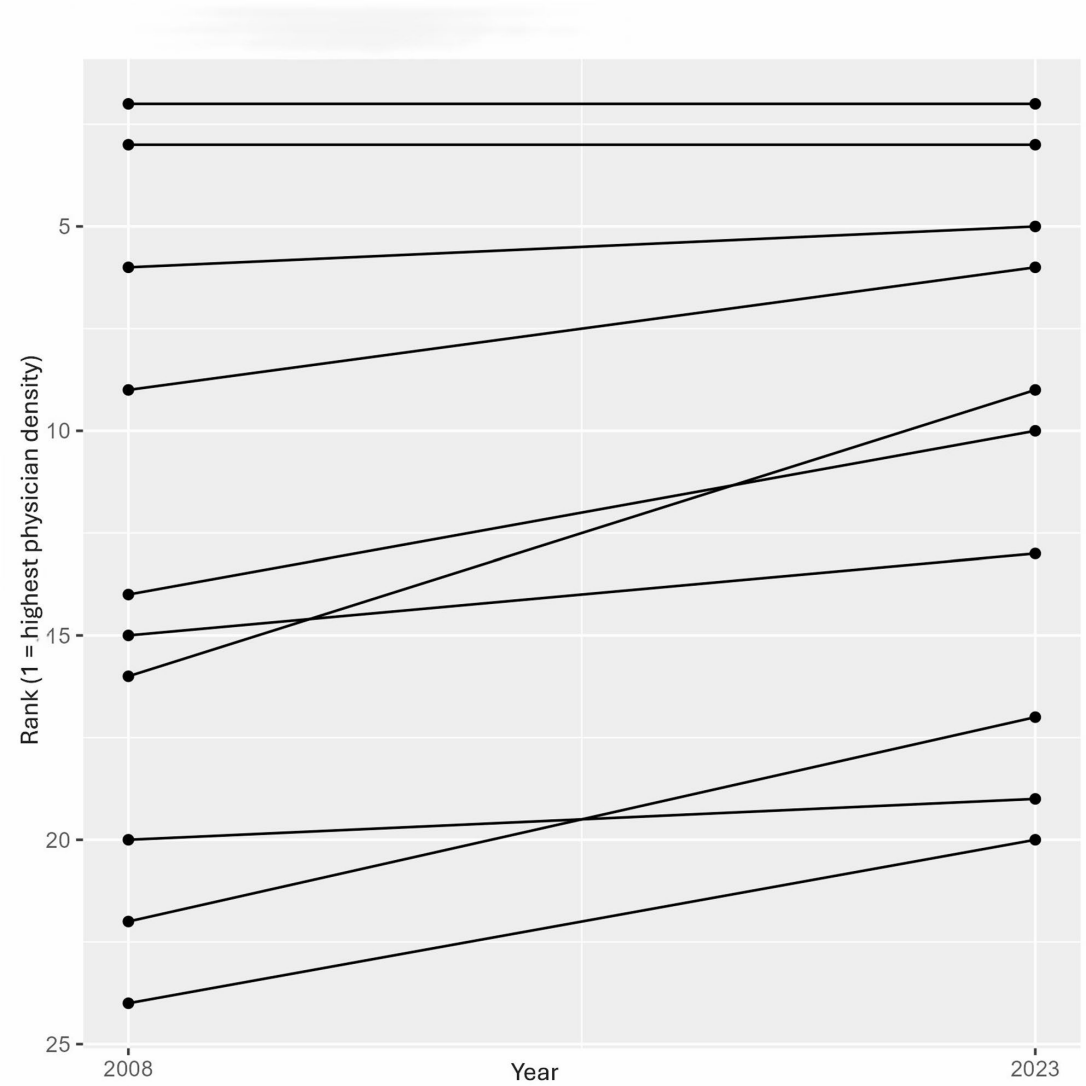
Regional rank mobility in physician density, 2008–2023. The figure illustrates changes in regional rankings based on physician density over time, highlighting relative mobility and persistence across regions

Rank-mobility analysis revealed meaningful positional changes among several regions over time (Fig. 4). Detailed rank-mobility results and local clustering patterns (LISA) are presented in the Supplementary Material (Figure S1).

Some mid-performing regions experienced upward mobility in physician density rankings, indicating partial catch-up in service capacity. However, regions located in the eastern and southeastern parts of the country remained persistently at the lower end of the distribution, underscoring the durability of the west–east gradient in physician availability.

### Summary of key findings

Overall, the results indicate that physician supply in Türkiye has expanded substantially and that regional disparities have narrowed through a process of β-convergence. Nevertheless, convergence has been incomplete and spatially uneven, with limited evidence of spillover effects between neighbouring regions. The combined evidence from β-convergence, σ-convergence, and spatial analyses suggests that while relative disparities have decreased, structural regional differences in physician availability persist. These findings suggest that while national workforce growth has improved aggregate service capacity, regional imbalances in physician availability continue to shape health service delivery.

Additional robustness analyses by region group, time period, and income regime are reported in the Supplementary Material (Tables S1–S10; Figures S1–S5).

## Discussion

This study examined long-term regional dynamics in physician workforce distribution in Türkiye from a health services perspective, conceptualising physician density as an indicator of regional service capacity. By integrating β-convergence analysis with spatial econometric methods, the study provides evidence on whether historically underserved regions have narrowed physician-related service gaps over time. Three principal findings emerge: (i) regional disparities in physician supply have narrowed through a statistically significant process of β-convergence, (ii) convergence has been uneven and strongly conditioned by regional economic and infrastructural factors, and (iii) spatial spillovers between neighbouring regions have played a limited role in redistributing physician-related service capacity.

### Convergence in physician supply and service capacity

The presence of statistically significant β-convergence indicates that regions with initially lower physician density experienced relatively faster growth over the study period. From a health services perspective, this finding suggests a gradual reduction in disparities in service capacity rather than full equalisation. Consistent with the Results, this pattern reflects relative catch-up dynamics rather than rapid structural convergence, particularly given persistent regional constraints. Similar patterns have been documented in several European and middle-income country contexts, where increases in overall physician supply have contributed to partial reductions in regional disparities over time [44–46].

However, convergence should not be interpreted as the elimination of inequality. Despite measurable catch-up, substantial differences in physician availability persisted, particularly between western and eastern regions of Türkiye. This coexistence of convergence and persistent disparities highlights that convergence processes can operate alongside structural inequalities. This finding is consistent with international evidence showing that workforce expansion alone is insufficient to overcome entrenched regional disadvantages affecting service delivery [47, 48]. Physician density captures only one dimension of service capacity; broader structural conditions continue to shape the effectiveness and sustainability of workforce distribution.

### Economic and infrastructural determinants of physician distribution

Regional economic capacity and health infrastructure emerged as key correlates of physician density, underscoring their central role in shaping service availability. Regions with higher income levels and greater hospital bed capacity were more likely to exhibit higher physician density, reinforcing existing advantages in service provision. These findings align closely with prior studies demonstrating that physician location decisions are influenced by regional labour market conditions, facility availability, and professional practice environments [49–51].

From a health services planning standpoint, these results highlight the limitations of workforce policies implemented in isolation. In particular, the findings suggest that improvements in physician supply are closely linked to broader regional conditions, including economic development and infrastructure. Allocation strategies that do not account for local economic and infrastructural contexts may struggle to achieve durable improvements in service capacity. Integrating physician workforce planning with regional development policies and infrastructure investment appears important for sustaining convergence and improving access in underserved areas [52, 53].

### Spatial dynamics

Spatial analyses revealed limited evidence of strong geographic spillover effects. Both global Moran’s I statistics and spatial econometric results indicate weak spatial dependence in physician distribution. This finding suggests that improvements in physician availability tend to remain largely region-specific rather than diffusing across neighbouring regions. Comparable patterns have been observed in other middle-income settings, where improvements in health workforce resources remain geographically concentrated [54, 55].

For health systems, this implies that reliance on indirect spillover effects is unlikely to substantially reduce regional disparities. Targeted, region-specific interventions are therefore required to address persistent service gaps.

### Regional mobility and persistent gradients

Rank-mobility analysis indicated some upward movement among mid-performing regions, suggesting partial relative catch-up in service capacity. However, regions at the lower end of the distribution—particularly in eastern and southeastern Türkiye—remained persistently disadvantaged. This enduring gradient is consistent with earlier studies documenting long-standing regional inequalities in physician distribution despite national reforms [56, 57]. Persistent low physician availability in these regions may have implications for access to care and service continuity. International evidence links workforce shortages to unmet health needs and access challenges, particularly among vulnerable populations [58, 59].

### Policy implications for health service planning

The findings carry several implications for health service planning and workforce policy. First, national increases in physician supply can support convergence but are unlikely to ensure balanced service delivery without complementary regional strategies.

Second, economic development and infrastructure investment appear central to strengthening service capacity and should be considered alongside workforce policies. Third, the limited role of spatial spillovers suggests that geographically targeted interventions may be more effective than uniform national approaches [56, 60, 61].

Overall, these findings suggest that translating workforce growth into equitable service capacity requires integrated policies that address both supply expansion and regional structural conditions.

### Strengths and limitations

This study has several strengths. The use of a long-term balanced panel enabled assessment of dynamic regional trends rather than static disparities. The combination of convergence analysis with spatial econometric methods provided a comprehensive evaluation of both temporal and geographic dimensions of physician distribution. Moreover, reliance on official and publicly available data enhances transparency and reproducibility.

Several limitations should also be acknowledged. First, the use of aggregated NUTS-2 regional data may mask within-region heterogeneity and local disparities in physician distribution. Second, physician density, while widely used, does not capture differences in productivity, specialty composition, or quality of care. Third, the ecological design limits causal inference and the ability to draw conclusions at the individual level. Finally, although multiple model specifications were employed, unobserved confounding factors cannot be entirely ruled out.

Future research could incorporate more granular data, specialty-specific indicators, or patient-level outcomes to further clarify the relationship between workforce distribution and health service performance.

### Ethics declaration

Not applicable.

### Funding

This research did not receive any specific grant from funding agencies in the public, commercial, or not-for-profit sectors.

## Conclusions

This study provides longitudinal and spatial evidence on regional dynamics in physician workforce distribution in Türkiye, framing physician density as a core indicator of health service capacity. The findings demonstrate that, although regional disparities in physician supply have narrowed over time through a process of convergence, this process has been uneven and spatially fragmented. Persistent regional gradients—particularly between western and eastern regions—continue to shape the distribution of health service capacity.

From a health services perspective, the results indicate that national increases in physician supply can contribute to aggregate improvements in service availability but are insufficient to ensure balanced regional service delivery. Economic conditions and health infrastructure play a decisive role in shaping physician availability, while spatial spillovers across neighbouring regions remain limited. These patterns suggest that workforce gains tend to remain locally contained rather than diffusing geographically.

For health system planners, the findings highlight the importance of integrating workforce policies with region-specific economic and infrastructural strategies. Policies that focus solely on increasing physician numbers may achieve convergence at the national level but fall short of addressing persistent service capacity gaps at the regional level. Strengthening health service delivery in underserved regions is therefore likely to require coordinated investments in infrastructure, supportive professional environments, and locally tailored workforce incentives.

Overall, this study underscores the value of longitudinal and spatially explicit approaches for understanding health service capacity and workforce dynamics. Such approaches can inform more effective and geographically responsive planning strategies aimed at achieving resilient and equitable health service delivery systems in Türkiye and comparable middle-income settings.

## Supplementary Information

Below is the link to the electronic supplementary material.

Supplementary Material 1

Supplementary Material 2

Supplementary Material 3

Supplementary Material 4

Supplementary Material 5

Supplementary Material 6

Supplementary Material 7

Supplementary Material 8

Supplementary Material 9

Supplementary Material 10

Supplementary Material 11

Supplementary Material 12

Supplementary Material 13

Supplementary Material 14

Supplementary Material 15

## Data Availability

The data used in this study are derived from publicly available sources, including official national health statistics, population statistics, and regional economic indicators. All data sources are cited in the manuscript. The datasets analysed during the current study are available from the corresponding author upon reasonable request.

## Abbreviations

NUTS: Nomenclature of Territorial Units for Statistics
SDM: Spatial Durbin Model
GDP: Gross Domestic Product
TWFE: Two-Way Fixed Effects
