# Supplementary figures and images for: Regional convergence and spatial dynamics of physician workforce distribution across regions in Türkiye (2008–2023)
Source: BMC Health Serv Res. 2026 Apr 24;26:818. doi: 10.1186/s12913-026-14519-w (PMC13267293; doi:10.1186/s12913-026-14519-w)

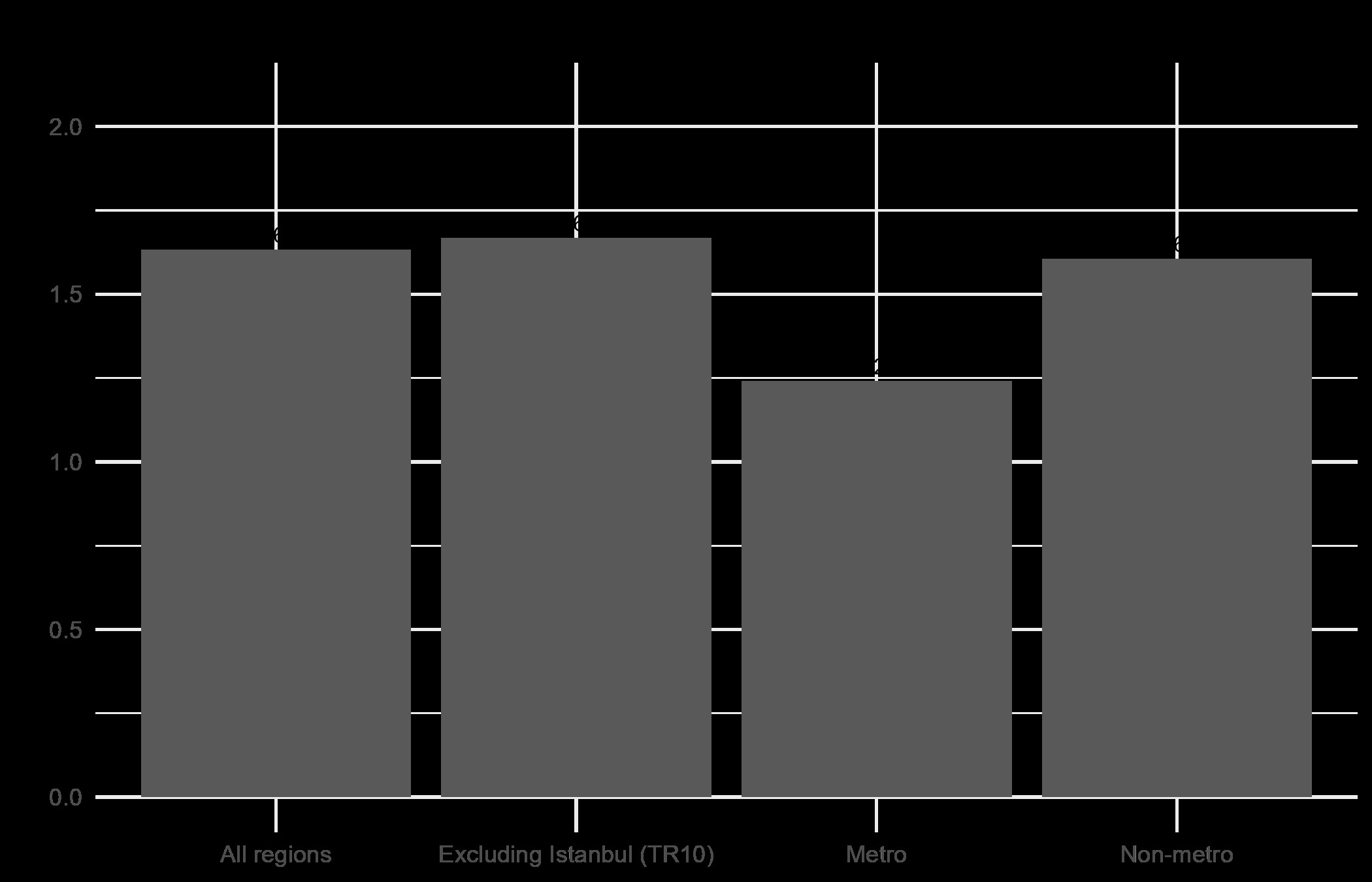

Supplement: Supplementary file 1 — Supplementary Material 1 [file 12913_2026_14519_MOESM1_ESM.jpg]

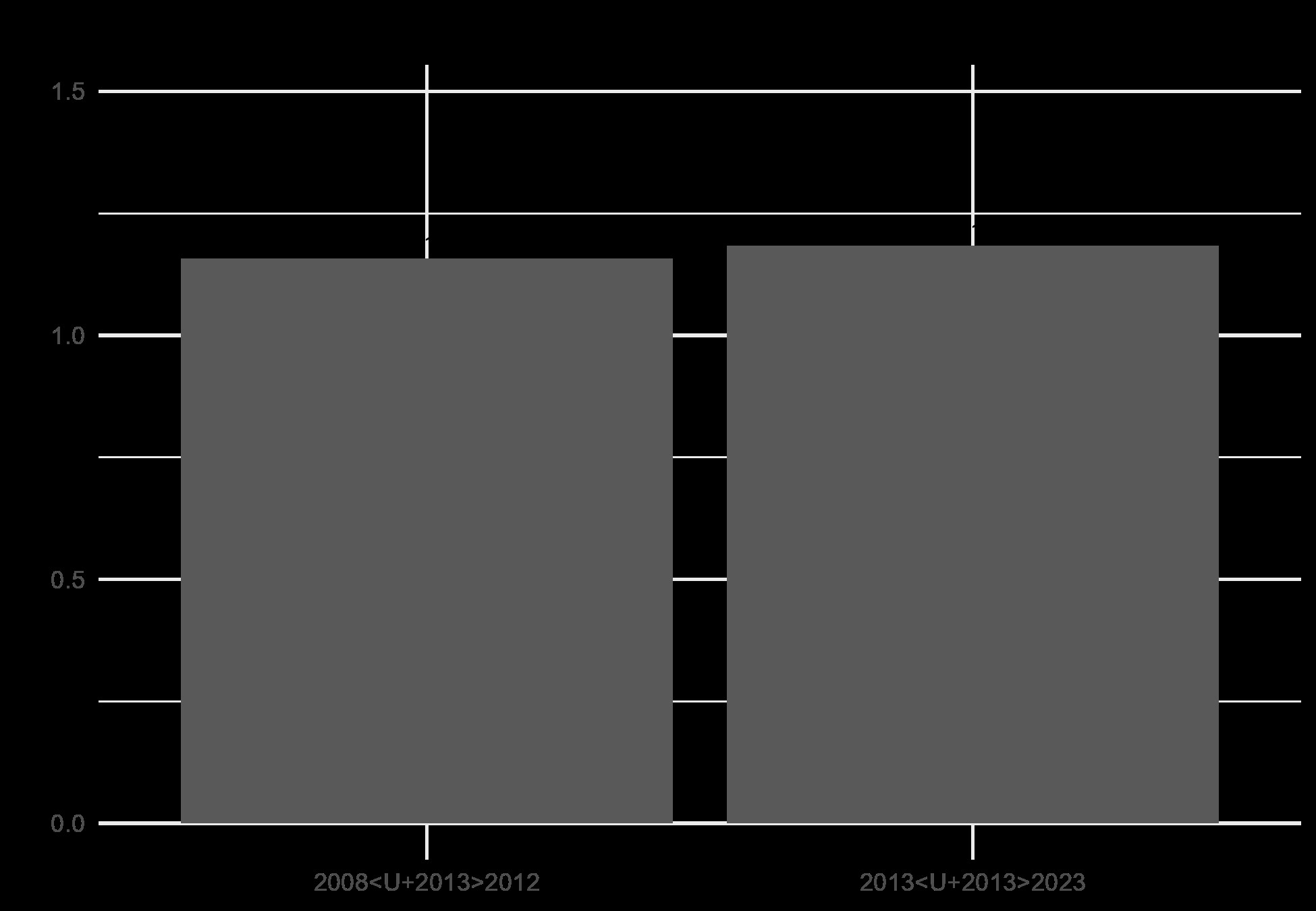

Supplement: Supplementary file 2 — Supplementary Material 2 [file 12913_2026_14519_MOESM2_ESM.jpg]

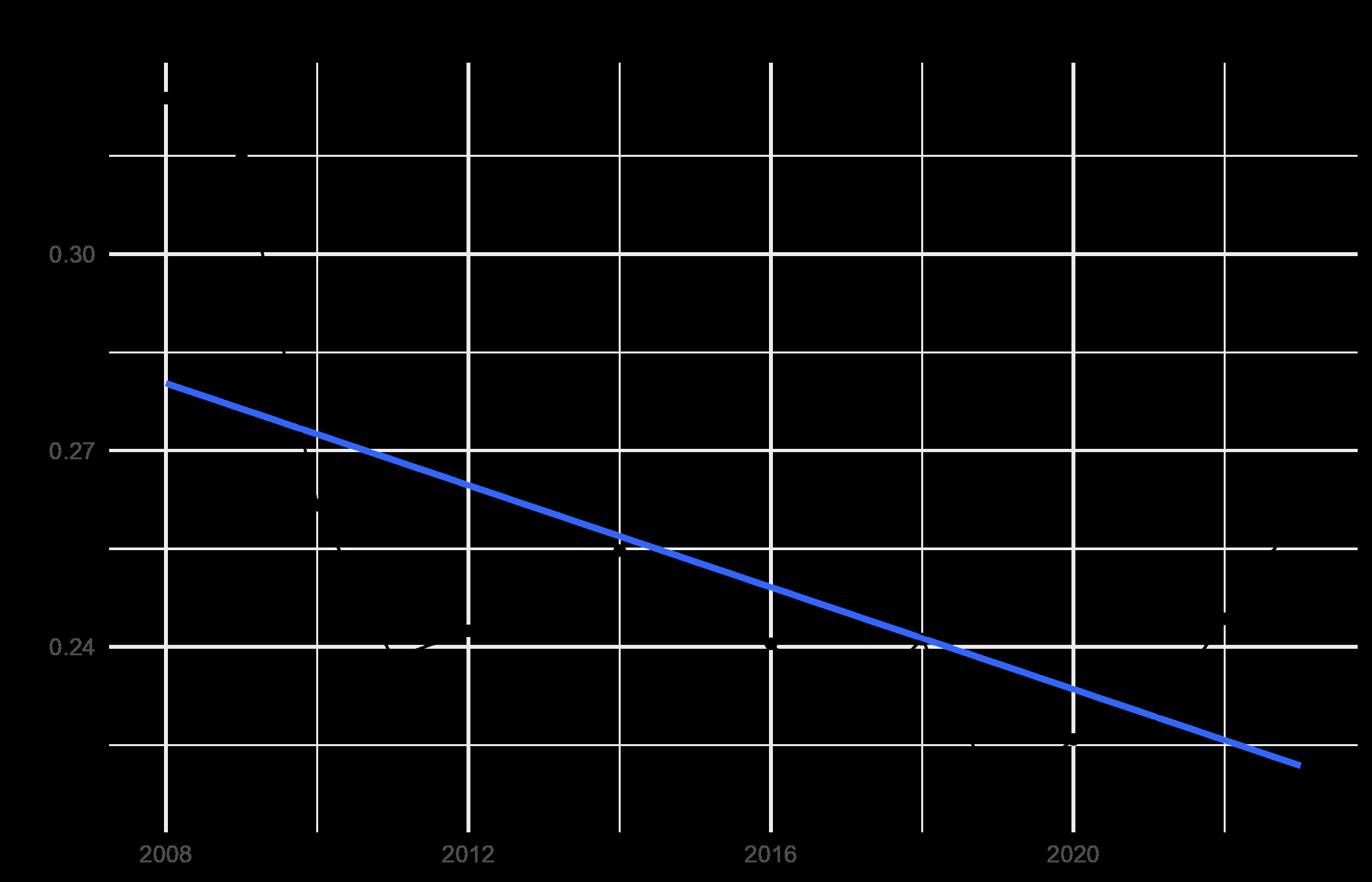

Supplement: Supplementary file 3 — Supplementary Material 3 [file 12913_2026_14519_MOESM3_ESM.jpg]

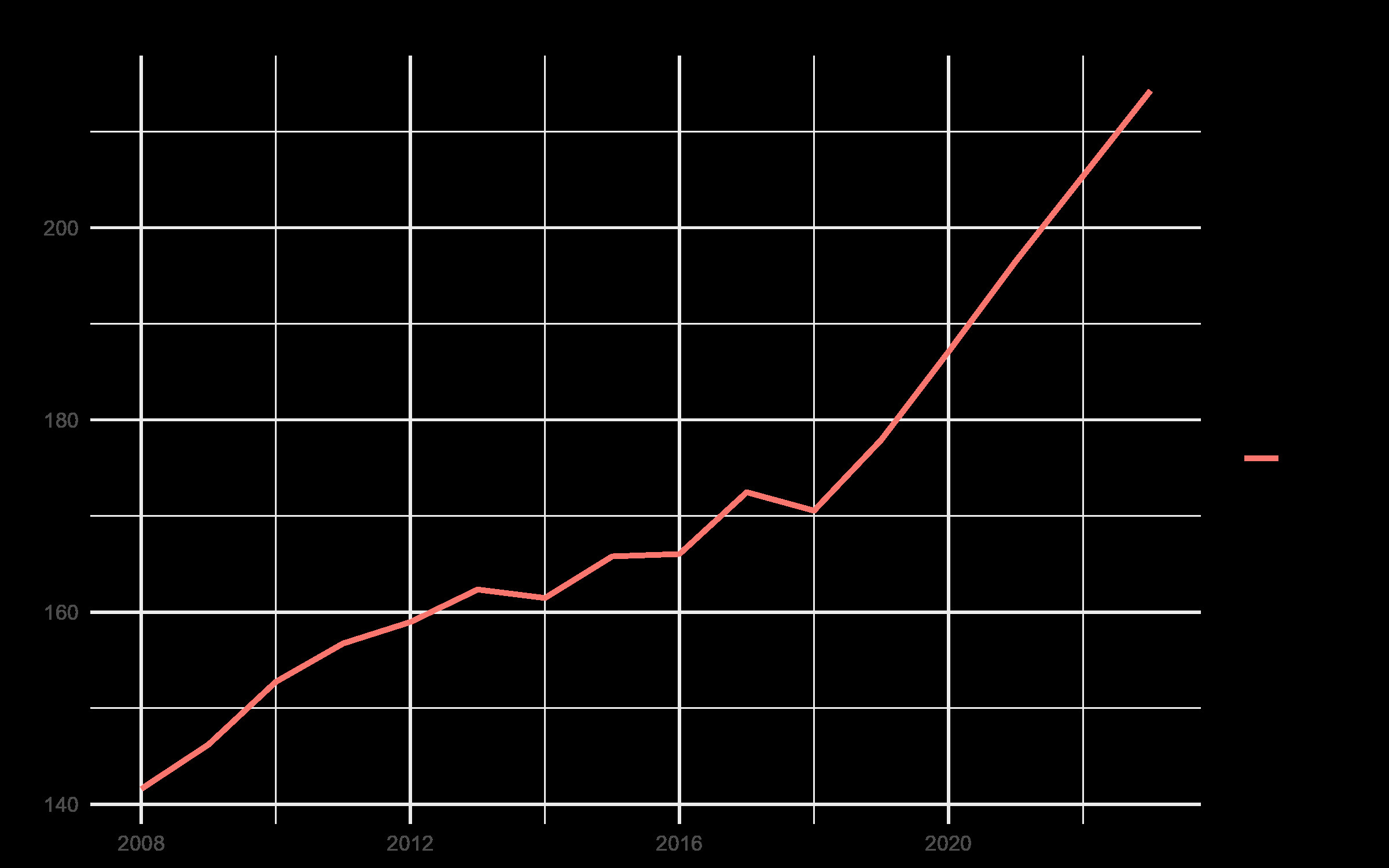

Supplement: Supplementary file 4 — Supplementary Material 4 [file 12913_2026_14519_MOESM4_ESM.jpg]

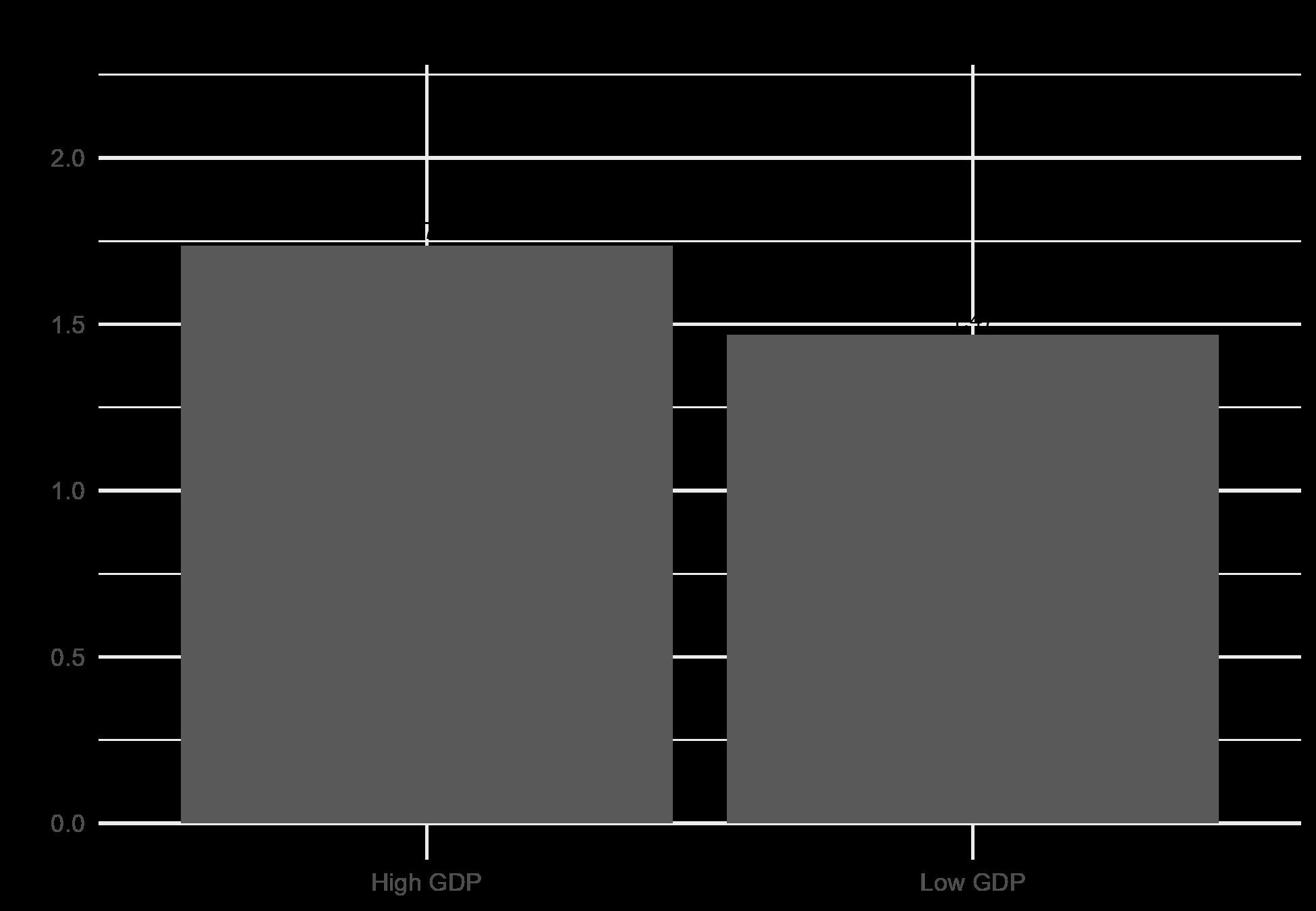

Supplement: Supplementary file 5 — Supplementary Material 5 [file 12913_2026_14519_MOESM5_ESM.jpg]
