# Supplementary material for: Regional convergence and spatial dynamics of physician workforce distribution across regions in Türkiye (2008–2023)
Source: BMC Health Serv Res. 2026 Apr 24;26:818. doi: 10.1186/s12913-026-14519-w (PMC13267293; doi:10.1186/s12913-026-14519-w)
Supplement: Supplementary file 6 — Supplementary Material 6 [file 12913_2026_14519_MOESM6_ESM.docx]

| spec | beta | se | p | lambda | half_life |
| --- | --- | --- | --- | --- | --- |
| Metro | -0.5584883210838186 | 0.09919466119548039 | 1.830679436066597e-6 | 0.5584883210838186 | 1.2411131162327689 |
| Non-metro | -0.4318294808977469 | 0.07226213633026361 | 6.699369088447063e-9 | 0.4318294808977469 | 1.6051409438719537 |
| Excluding Istanbul (TR10) | -0.41559994051853644 | 0.08589928955812581 | 2.0092537319332697e-6 | 0.41559994051853644 | 1.6678230985671418 |
| All regions | -0.4243353220239274 | 0.07831354100629175 | 1.1288059967820935e-7 | 0.4243353220239274 | 1.6334892350084873 |
