# Supplementary material for: Regional convergence and spatial dynamics of physician workforce distribution across regions in Türkiye (2008–2023)
Source: BMC Health Serv Res. 2026 Apr 24;26:818. doi: 10.1186/s12913-026-14519-w (PMC13267293; doi:10.1186/s12913-026-14519-w)
Supplement: Supplementary file 7 — Supplementary Material 7 [file 12913_2026_14519_MOESM7_ESM.docx]

| spec | beta | se | p | lambda | half_life |
| --- | --- | --- | --- | --- | --- |
| 2008<U+2013>2012 | -0.5985251443540901 | 0.10857254794075172 | 5.374867372986663e-7 | 0.5985251443540901 | 1.1580919984706213 |
| 2013<U+2013>2023 | -0.5856160693303956 | 0.14503796454689247 | 7.445254998583483e-5 | 0.5856160693303956 | 1.1836204927786609 |
