# Supplementary material for: Regional convergence and spatial dynamics of physician workforce distribution across regions in Türkiye (2008–2023)
Source: BMC Health Serv Res. 2026 Apr 24;26:818. doi: 10.1186/s12913-026-14519-w (PMC13267293; doi:10.1186/s12913-026-14519-w)
Supplement: Supplementary file 8 — Supplementary Material 8 [file 12913_2026_14519_MOESM8_ESM.docx]

t test of coefficients:

Estimate Std. Error t value Pr(>|t|)

(Intercept) 8.101203 13.925241 0.582 0.570

year -0.003895 0.006915 -0.563 0.582
