# Supplementary material for: Regional convergence and spatial dynamics of physician workforce distribution across regions in Türkiye (2008–2023)
Source: BMC Health Serv Res. 2026 Apr 24;26:818. doi: 10.1186/s12913-026-14519-w (PMC13267293; doi:10.1186/s12913-026-14519-w)
Supplement: Supplementary file 9 — Supplementary Material 9 [file 12913_2026_14519_MOESM9_ESM.docx]

| club | nuts2 |
| --- | --- |
| Club_1 | TR51 |
| Club_1 | TR31 |
| Club_1 | TR10 |
| Club_1 | TR61 |
| Club_1 | TR52 |
| Club_1 | TRA1 |
| Club_1 | TR72 |
| Club_1 | TRB1 |
| Club_1 | TR90 |
| Club_1 | TR83 |
| Club_1 | TR62 |
| Club_1 | TR32 |
| Club_1 | TR42 |
| Club_1 | TR21 |
| Club_1 | TR81 |
| Club_1 | TR41 |
| Club_1 | TR22 |
| Club_1 | TR33 |
| Club_1 | TRC1 |
| Club_1 | TR63 |
| Club_1 | TR71 |
| Club_1 | TR82 |
| Club_1 | TRC2 |
| Club_1 | TRB2 |
| Club_1 | TRA2 |
| Club_1 | TRC3 |
