# Supplementary material for: Regional convergence and spatial dynamics of physician workforce distribution across regions in Türkiye (2008–2023)
Source: BMC Health Serv Res. 2026 Apr 24;26:818. doi: 10.1186/s12913-026-14519-w (PMC13267293; doi:10.1186/s12913-026-14519-w)
Supplement: Supplementary file 10 — Supplementary Material 10 [file 12913_2026_14519_MOESM10_ESM.docx]

| spec | b_hat | t_stat |
| --- | --- | --- |
| All regions | -0.9887757584579713 | -9.071714806962747 |
