# Supplementary material for: Regional convergence and spatial dynamics of physician workforce distribution across regions in Türkiye (2008–2023)
Source: BMC Health Serv Res. 2026 Apr 24;26:818. doi: 10.1186/s12913-026-14519-w (PMC13267293; doi:10.1186/s12913-026-14519-w)
Supplement: Supplementary file 11 — Supplementary Material 11 [file 12913_2026_14519_MOESM11_ESM.docx]

| club | n | mean_2023 |
| --- | --- | --- |
| Club_1 | 26 | 214.24891848208827 |
