# Supplementary material for: Regional convergence and spatial dynamics of physician workforce distribution across regions in Türkiye (2008–2023)
Source: BMC Health Serv Res. 2026 Apr 24;26:818. doi: 10.1186/s12913-026-14519-w (PMC13267293; doi:10.1186/s12913-026-14519-w)
Supplement: Supplementary file 12 — Supplementary Material 12 [file 12913_2026_14519_MOESM12_ESM.docx]

| group | beta | se | p | lambda | half_life |
| --- | --- | --- | --- | --- | --- |
| Low GDP | -0.4721406907261104 | 0.07378053544620035 | 1.6079680511421914e-9 | 0.4721406907261104 | 1.4680945620127477 |
| High GDP | -0.3992348570941488 | 0.06895633889770532 | 3.593356260665827e-8 | 0.3992348570941488 | 1.7361890332047965 |
