# Supplementary material for: Regional convergence and spatial dynamics of physician workforce distribution across regions in Türkiye (2008–2023)
Source: BMC Health Serv Res. 2026 Apr 24;26:818. doi: 10.1186/s12913-026-14519-w (PMC13267293; doi:10.1186/s12913-026-14519-w)
Supplement: Supplementary file 13 — Supplementary Material 13 [file 12913_2026_14519_MOESM13_ESM.docx]

| nuts2 | 2008 | 2023 | delta |
| --- | --- | --- | --- |
| TR10 | 178.7 | 297.9 | 119.3 |
| TR90 | 125.7 | 225.1 | 99.4 |
| TR22 | 103.7 | 201.5 | 97.7 |
| TR61 | 167.3 | 263 | 95.7 |
| TRA1 | 142.6 | 237.1 | 94.5 |
| TR52 | 149 | 243 | 94 |
| TR83 | 132.9 | 214.1 | 81.2 |
| TR72 | 147.5 | 227.9 | 80.4 |
| TR33 | 118.1 | 198.2 | 80.1 |
| TR42 | 132.8 | 208.7 | 75.9 |
| TRC1 | 105.5 | 180.6 | 75.1 |
| TR63 | 97 | 170.5 | 73.5 |
| TR31 | 235.4 | 308.9 | 73.5 |
| TR62 | 139.7 | 212.3 | 72.6 |
| TR81 | 136.2 | 204.4 | 68.2 |
| TR21 | 140.5 | 208.1 | 67.6 |
| TR41 | 137.8 | 204.3 | 66.5 |
| TR32 | 145.3 | 211.2 | 65.9 |
| TRB1 | 165.1 | 226 | 60.9 |
| TR82 | 110.3 | 166.1 | 55.8 |
| TRA2 | 80.8 | 135.4 | 54.6 |
| TRC3 | 79.9 | 131.4 | 51.5 |
| TRB2 | 99 | 147.5 | 48.6 |
| TR71 | 120.5 | 168.4 | 47.9 |
| TRC2 | 104.3 | 151.1 | 46.9 |
| TR51 | 386 | 427.6 | 41.6 |
