# Supplementary material for: Regional convergence and spatial dynamics of physician workforce distribution across regions in Türkiye (2008–2023)
Source: BMC Health Serv Res. 2026 Apr 24;26:818. doi: 10.1186/s12913-026-14519-w (PMC13267293; doi:10.1186/s12913-026-14519-w)
Supplement: Supplementary file 14 — Supplementary Material 14 [file 12913_2026_14519_MOESM14_ESM.docx]

| var | type | effect | z | p |
| --- | --- | --- | --- | --- |
| 1 | direct | 1.7268100933205148e-5 | NA | NA |
| 2 | direct | -2.2105858888691983 | NA | NA |
| 3 | direct | 2.273740904076843 | NA | NA |
| 1 | indirect | 1.3987294818845857e-6 | NA | NA |
| 2 | indirect | -9.959407085800787 | NA | NA |
| 3 | indirect | -0.556102006933672 | NA | NA |
| 1 | total | 1.8666830415089733e-5 | NA | NA |
| 2 | total | -12.169992974669986 | NA | NA |
| 3 | total | 1.7176388971431709 | NA | NA |
