# Supplementary material for: Regional convergence and spatial dynamics of physician workforce distribution across regions in Türkiye (2008–2023)
Source: BMC Health Serv Res. 2026 Apr 24;26:818. doi: 10.1186/s12913-026-14519-w (PMC13267293; doi:10.1186/s12913-026-14519-w)
Supplement: Supplementary file 15 — Supplementary Material 15 [file 12913_2026_14519_MOESM15_ESM.docx]

spec beta lambda half_life

FE

(TWFE, -

DK) 0.424335322023930.424335322023931.6334892350085 FE no-

controls -

(DK) 0.393535420990130.393535420990131.7613336528031 FE

weighted

(fixest,

2way -

cluster) 0.489157214807320.489157214807321.4170233200649
